# Nonvitamin K Antagonist Oral Anticoagulants Versus Warfarin in Atrial Fibrillation Patients and Risk of Dementia: A Nationwide Propensity‐Weighted Cohort Study

**DOI:** 10.1161/JAHA.118.011358

**Published:** 2019-05-29

**Authors:** Mette Søgaard, Flemming Skjøth, Martin Jensen, Jette Nordstrøm Kjældgaard, Gregory Y. H. Lip, Torben Bjerregaard Larsen, Peter Brønnum Nielsen

**Affiliations:** ^1^ Department of Cardiology Aalborg University Hospital Aalborg Denmark; ^2^ Aalborg Thrombosis Research Unit Department of Clinical Medicine Faculty of Health Aalborg University Aalborg Denmark; ^3^ Unit for Clinical Biostatistics Aalborg University Hospital Aalborg Denmark; ^4^ Liverpool Centre for Cardiovascular Science University of Liverpool and Liverpool Heart & Chest Hospital Liverpool United Kingdom; ^5^ Aalborg Thrombosis Research Unit Department of Clinical Medicine Aalborg University Aalborg Denmark

**Keywords:** anticoagulants, atrial fibrillation, dementia, direct oral anticoagulant, warfarin, Atrial Fibrillation

## Abstract

**Background:**

It is unclear whether nonvitamin K antagonist oral anticoagulants (NOACs) can mitigate dementia development in atrial fibrillation. We compared dementia development among users of NOACs or warfarin in patients with atrial fibrillation with no prior neurological diagnoses.

**Methods and Results:**

We conducted a Danish nationwide cohort study including 33 617 new oral anticoagulant users with nonvalvular atrial fibrillation, of which 11 052 were aged 60 to 69 years, 13 237 were aged 70 to 79 years, and 9238 were aged 80 years and older. To exclude prevalent non‐oral anticoagulants–associated dementia, we considered the at‐risk population of patients alive and free of dementia at 180 days following inclusion. We compared rates of new‐onset dementia by age and treatment regimen using inverse probability of treatment weighting to account for confounding. Approximately 60% of patients were NOAC users and 40% were warfarin users. Mean follow‐up was 3.4 years. Dementia occurred in 41 patients aged 60 to 69 years, 276 patients aged 70 to 79 years, and 441 patients aged 80 years and older. Relative to warfarin users, dementia rates were nonsignificantly lower among NOAC users aged 60 to 69 years (0.11 events/100 person‐years versus 0.12 events/100 person‐years; weighted hazard ratio, 0.92 [95% CI, 0.48–1.72]) and NOAC users aged 70 to 79 years (0.64 events/100 person‐years versus 0.78 events/100 person‐years; weighted hazard ratio, 0.86 [95% CI, 0.68–1.09]), whereas NOACs were associated with significantly higher dementia rates (2.16 events/100 person‐years versus 1.70 events/100 person‐years; weighted hazard ratio, 1.31 [95% CI, 1.07–1.59]) in patients 80 years and older.

**Conclusions:**

This nationwide cohort of patients with atrial fibrillation revealed no clinically meaningful difference in dementia development between users of NOACs or warfarin apart from a higher risk in NOAC users 80 years and older, which may relate to residual confounding from selective prescribing and unobserved comorbidities.

**Clinical Perspective**

**What Is New?**

- Growing evidence suggests an association between atrial fibrillation (AF) and dementia. It has been hypothesized that nonvitamin K antagonist oral anticoagulants (OACs) could play a role in reducing the risk of cognitive impairment and dementia in patients with AF, but the available evidence is sparse and equivocal.
- This study investigated a large well‐defined, nationwide, population‐based cohort of patients with nonvalvular AF initiating OAC treatment during 2011 through 2016 and compared rates of new‐onset dementia by age and treatment regimen (nonvitamin K antagonist OACs versus warfarin).
- Among patients with AF with no prior neurological disorders, rates of new‐onset dementia was nonsignificantly different among patients initiating nonvitamin K antagonist OAC therapy versus warfarin.

**What Are the Clinical Implications?**

- In the absence of disease‐modifying treatments for most forms of dementia, any means to prevent or delay its onset is of major clinical and social importance.
- This real‐world observational study suggests no difference of nonvitamin K antagonist OACs over well‐managed warfarin therapy.
- However, potential residual confounding related to selective prescribing and unobserved comorbidities warrants cautious clinical interpretation, and further studies, including randomized trials, are needed to fully investigate the impact of OAC treatment in AF on dementia development and progression.

## Introduction

Atrial fibrillation (AF) is the most common cardiac arrhythmia and is associated with up to a 5‐fold increased risk of stroke.^1^, ^2^ Owing to the increasing life expectancy, both prevalence and incidence rates of AF are projected to increase over the coming decades. Substantial increases in rates of dementia are also expected given the strong correlation with age.^3^

Dementia is a condition that impairs memory and other cognitive abilities, causing significant disability. Growing evidence suggests an association between AF and dementia even if no clinical strokes have occurred.^4^, ^5^, ^6^, ^7^, ^8^ Nevertheless, the pathological mechanism underlying the association between AF and dementia is unclear. Plausible mechanisms include AF‐related microemboli and microbleeds and shared risk factors for AF and dementia such as coronary artery disease, hypertension and hypotension, heart failure, diabetes mellitus, and age.^9^

Oral anticoagulant (OAC) therapy with vitamin K antagonists (eg, warfarin) or with nonvitamin K antagonist OACs (NOACs) is central for stroke prevention in AF.^10^ While warfarin traditionally has been the mainstay of treatment, meticulous dosage titration may be necessary to obtain an anticoagulation effect within the target therapeutic range (TTR). If not achieved, the treatment may entail periods of supratherapeutic and subtherapeutic effects from warfarin, which can lead to microemboli and microbleeds.^11^ Indeed, time outside the TTR in patients with AF receiving warfarin has been associated with increased risk of dementia^6^, ^11^

Compared with warfarin, NOACs have a favorable risk‐benefit profile with significant reductions in stroke, in particular a reduction in intracerebral hemorrhage.^12^ Because of the more predictable pharmacokinetics, NOACs may mitigate the challenges with TTR from dose titration. It is therefore plausible that NOACs in comparison with warfarin may prevent silent (or undetected) infarcts and microbleeds, and consequently may be able to slow the cognitive decline attributable to AF and AF risk factors. Nevertheless, randomized controlled trials that have assessed the efficacy and safety of NOAC versus warfarin did not include cognitive function as outcome, and data comparing rates of dementia among patients with AF treated with warfarin or NOAC are scarce.^13^, ^14^

The purpose of this study was to study the effect of an anticoagulant treatment regimen (NOAC or warfarin) on the risk of new‐onset dementia in clinical practice using a nationwide Danish cohort of OAC‐naive patients with AF with no prior neurological diagnoses.

## Methods

The source population for this cohort study comprised all citizens of Denmark encompassing 5.6 million inhabitants. Health care in Denmark is provided through a national tax‐funded system. As a result, data on diagnoses and prescription claims are compiled in longitudinal national registries allowing true nationwide population‐based studies. The present study utilized linkage between 3 nationwide medical registries. The first was the National Patient Register^15^ which encompasses information from all inpatient stays and outpatient visits at Danish hospitals, including dates of hospitalization and discharge, outpatient clinic visits, surgical procedures, and discharge *International Classification of Diseases* (*ICD*) diagnoses since 1977. The second registry was the National Prescription Register,^16^ which holds data on all prescription purchases by Danish residents since 1995. Data include the patients’ civil registration number, date of dispensing, and type and quantity of drug prescribed. The third registry was the Danish Civil Person Registry,^17^ which contains data on sex, date of birth, and vital and emigration status. We linked these registries using a unique 10‐digit personal registration number assigned to each Danish citizen at birth and to residents upon immigration.^17^

### Ethical Considerations and Data Availability

The study was approved by the Danish Data Protection Agency (reference 2015‐57‐0001). Registry studies do not require ethical approval in Denmark. The data were provided by the Danish Health Data Authority. Because of restrictions related to Danish law and protecting patient privacy, the combined set of data as used in this study can only be made available through the Danish Health Data Authority.^18^ This state organization holds the data used for this study. Researchers can apply for access to these data when the request is approved by the Danish Data Protection Agency.^19^

### Study Population

We identified all patients 60 years and older with a first‐time prescription redemption of apixaban (introduced December 10, 2012), dabigatran (introduced August 1, 2011), rivaroxaban (introduced February 1, 2012), or warfarin (since August 1, 2011, to match the NOAC treatment regimen) who had a previous hospital AF diagnosis or received an AF diagnosis within 30 days after prescription redemption. Patients with edoxaban treatment were not considered because of the late market entry in the study period. Study patient inclusion was terminated by December 31, 2016. We did not include patients with prior experience with any OAC in order to establish an OAC‐naive cohort, as was previously performed.^20^ We excluded patients who had not been residents in Denmark for at least 5 years before date of first OAC prescription redemption to ensure sufficient lookback time for diagnoses of prevalent dementia as well as comorbidities and medications. To focus on nonvalvular AF, we excluded patients with previous diagnoses indicating valvular AF (mitral stenosis or mechanical heart valves). We further excluded patients with another indication for OAC treatment (ie, history of venous thromboembolism within 90 days before prescription redemption). Finally, because we were interested in incident dementia events under OAC treatment exposure, we excluded patients with previous dementia diagnoses, use of antidementia medications, or diagnosis of mild cognitive impairment or an amnestic syndrome, which may represent prodromal dementia, and patients with previous neurological diagnoses including ischemic or hemorrhagic stroke, transient ischemic attack (TIA), or traumatic brain injury before first OAC prescription redemption.

### Definition of Landmark Population, Exposure, and Follow‐Up

To study exposure of OAC treatment and rates of dementia, we considered the at‐risk population of patients who had purchased a first prescription of an NOAC or warfarin who were alive and free of dementia at the landmark time point of 180 days following the date of OAC prescription purchase that led to inclusion in the study. The reasoning underlying this choice of landmark analysis was to exclude possible prevalent and non–OAC‐associated dementia cases, since dementia diagnosed shortly after the first OAC prescription is unlikely to be causally related to the treatment. We used recent anticoagulant purchase in the landmark population to determine patients’ anticoagulant therapy (NOAC versus warfarin). To ensure steady state OAC treatment, patients were required to fill at least 2 prescriptions of the same type of OAC during the 180‐day landmark period (“extended anticoagulant usage”), since the typical patient persisting with therapy would purchase anticoagulants at least twice during any 6‐month period.^21^ Patients with both warfarin and NOAC prescriptions during the landmark period were excluded. We subsequently followed patients from the 180‐day landmark until a diagnosis of dementia, emigration, death, or study end (March 23, 2018), whichever came first.

### Study End Points

The primary study outcome was a composite of incident dementia subtypes defined by hospital inpatient and outpatient clinic diagnoses of dementia recorded in the National Patient Registry from the 180‐day landmark. The secondary outcomes were the specific dementia subtypes (Alzheimer disease, vascular dementia, and other dementia). The hospital admission date or start date of outpatient clinic follow‐up was considered the date of dementia diagnosis. In the National Patient Registry, dementia diagnoses are available for hospital admissions since 1977 and for outpatient visits since 1995.^15^ The positive predictive value of inpatient and outpatient diagnoses of all‐cause dementia is 86% and 81% for Alzheimer disease.^22^

### Ascertainment of Comorbidity

Using the patients’ medical history since 1994 (introduction of *ICD*‐*Tenth Revision* in Denmark) and medication claims, we obtained information on comorbidities at the time of OAC prescription, which could possibly confound the causal association between choice of OAC treatment for stroke prevention in nonvalvular AF and risk of new‐onset dementia. Comorbidity information included cardiovascular and metabolic diseases, lifestyle‐related diseases, and information on depression and substance abuse. Table S1 provides information on all codes for diagnoses and medications.

We further combined covariate information into the Charlson Comorbidity Index,^23^ CHA_2_DS_2_VASc score^24^ as a measure of stroke risk, and HAS‐BLED score^25^ as a measure of bleeding risk (see score definitions in Tables S2 and S3).

### Statistical Analysis

We provided descriptive summaries of patient baseline characteristics at the time of index OAC prescription redemption and at the 180‐day landmark as proportions for discrete variables and means and SDs for continuous variables, and stratified according to treatment regimen. Because the risk of dementia is strongly associated with age, ie, the hazard is expected to change as a function of age, we conducted all analyses stratified by age group (categorized as 60–69 years, 70–79 years, and 80 years and older) at the time of the 180‐day landmark. To compare the risk of dementia among NOAC users with warfarin (reference) users within each age group, we calculated cause‐specific hazard ratios (HRs) using Cox regression models. Failure curves were used to depict how dementia risk evolved over time. Specifically, we used the Aalen‐Johansen estimator to calculate absolute risk of dementia in each age group, taking into account the competing risk of death.^24^ Study end points were examined using the full follow‐up period available.

To allow an unbiased comparison of NOAC users and warfarin users, we applied an inverse probability of treatment weighting approach. With this approach, stabilized weights were derived to obtain estimates representing population average treatment effects with optimal balance between the treatment populations within each age group.^26^, ^27^ We used generalized boosted models based on up to 10 000 regression trees to obtain the propensity for receiving a treatment allocation, as previously performed.^20^, ^28^ The covariates for the regression trees included indicators of comorbidity and concomitant medical treatment at the time of study inclusion (eg, first OAC prescription redemption) (see Table S4 for specification of variables). The examined treatment regimens should be contrasted on comparable populations and any patient must have positive probability for any treatment within all covariate strata (positivity assumption). We therefore required substantial overlap between the propensities for each treatment group to ensure exchangeability (under assumption of correct model specification), and inspected the distribution of weights to detect extreme values, which may indicate violation of the positivity assumption.^29^ In agreement with best methodological practice, this assumption was assessed by graphical inspection of the weight distributions.^30^ Additionally, balance between treatment populations was evaluated by standardized differences of all baseline covariates, using a threshold of 0.1 to indicate imbalance.^31^

### Sensitivity Analyses

We performed 4 sensitivity analyses to ascertain the robustness of our findings. First, given the assumed induction period in the development of dementia, we repeated the analyses excluding the initial 365 days following the date of OAC prescription (eg, using a 365‐day landmark). Second, because duration of AF may affect dementia development, we conducted an analysis stratified by incident versus nonincident AF. Third, to study potential selective prescribing of reduced‐dose NOACs to patients with prodromal symptoms of dementia, we conducted analyses stratified by NOAC dose (standard versus reduced dose); warfarin is only available in 2.5‐mg dose tablets in Denmark and dosed individually. Last, to evaluate the presence of residual confounding, we conducted analyses on “falsification outcomes,” ie, outcomes that a priori should not represent a causal effect of treatment.^32^ For this analysis, we used hospital diagnoses of pneumonia and diagnoses of urinary tract infection ascertained by prescriptions claim for trimethoprim, pivmecillinam, sulfamethizole, or nitrofurantoin, which are used specifically for urinary tract infection treatment in Denmark.^33^ We recalculated all weights in all supplementary and sensitivity analyses and inspected weight distributions and standardized differences. All standardized differences were below the 0.1 threshold, with exception of the estimates for renal dysfunction in the analyses of patients with reduced‐dose NOAC, where the standardized differences ranged from 0.19 to 0.26 for NOAC versus warfarin users stratified by age group.

Point estimates were reported with 95% CIs and a *P*<0.05 was considered statistically significant. All analyses were performed using STATA/MP version 15 (StataCorp) and R version 3.3.3 (The R Foundation).

## Results

**Figure 1:**
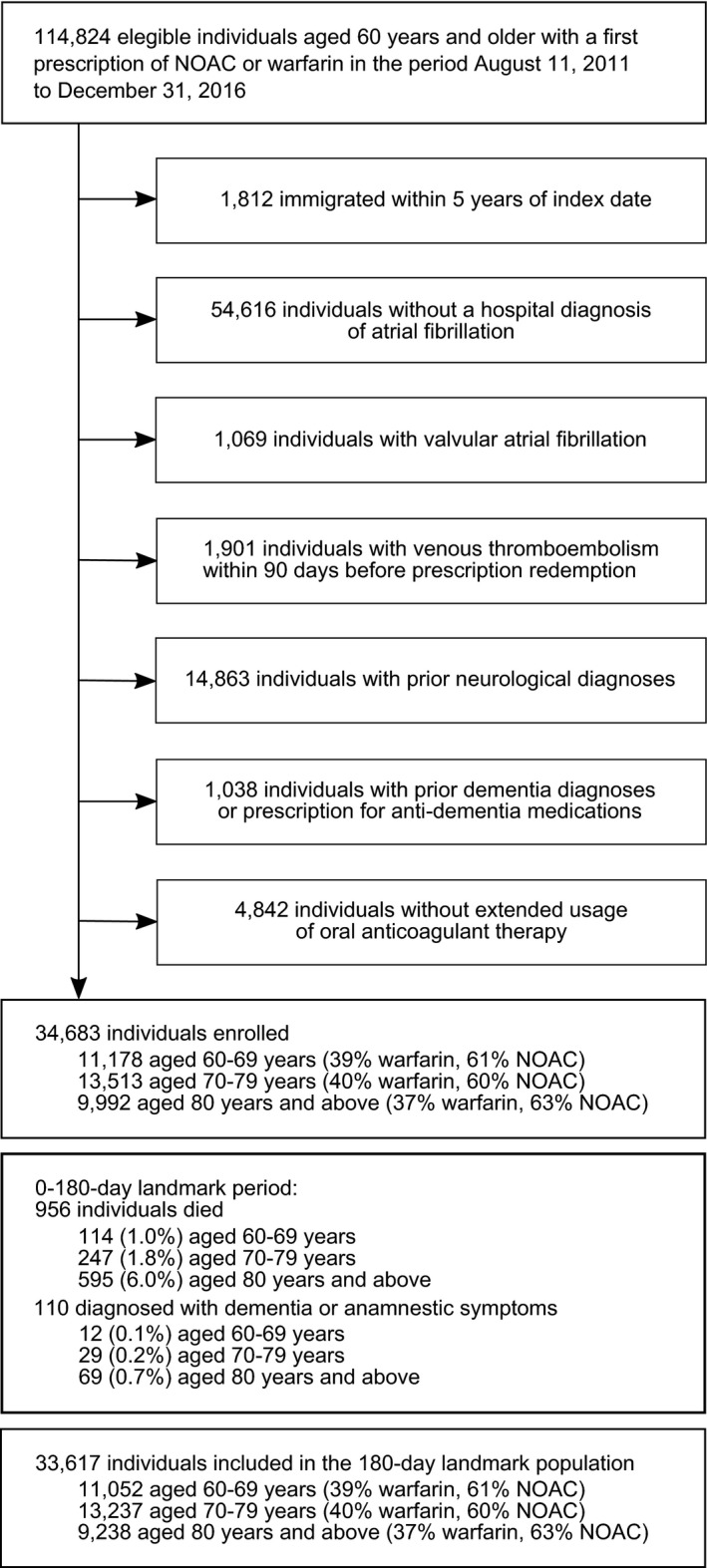
Flowchart of the study population. NOAC indicates nonvitamin K antagonist oral anticoagulant.

Between August 11, 2011, and December 31, 2016, we identified 114 824 new users of OAC treatment aged 60 years and older. After exclusions, the study population comprised 34 683 incident OAC users with hospital‐diagnosed AF. Of these, 11 178 (32%) were aged 60 to 69 years, 13 513 (39%) were 70 to 79 years, and 9992 (29%) were 80 years and older (Figure 1). Overall, ≈60% of patients were NOAC users and 40% were warfarin users, and this distribution was generally similar across the 3 age groups. Conversely, the proportion of NOAC users given a reduced dose increased considerably with increasing age, from 9.0% in patients aged 60 to 69 years, to 23.0% in patients aged 70 to 79 years and 73.7% in patients 80 years and older. Table 1 provides patient characteristics by age and treatment regimen at index OAC prescription before applying propensity weighting. Mean CHA_2_DS_2_‐VASc, HAS‐BLED, and Charlson Comorbidity Index scores increased with increasing age but varied little by treatment regimen. Recorded *ICD* codes of smoking‐, alcohol‐, or drug abuse–related diagnoses were infrequent across all age groups and treatment regimens.

**Table 1:** Patient Characteristics by Age at First OAC Prescription Redemption of OAC‐Naive Patients With AF (N=34 683)

Characteristic, % (No.)	60 to 69 y		70 to 79 y		80 y and Older	
	Warfarin	NOAC	Warfarin	NOAC	Warfarin	NOAC
No.	4332	6846	5387	8126	3653	6339
Median time from first AF diagnosis to first OAC prescription, d (IQR)	19 (4–546)	8 (2–438)	12 (4–230)	7 (3–136)	11 (5–162)	9 (4–144)
Dabigatran	…	46.1 (3156)	…	39.1 (3178)	…	32.8 (2078)
Apixaban	…	28.3 (1940)	…	32.2 (2618)	…	39.4 (2496)
Rivaroxaban	…	25.6 (1750)	…	28.7 (2330)	…	27.8 (1765)
Reduced‐dose NOAC	…	9.0 (614)	…	23.0 (1867)	…	73.7 (4673)
Hospital stay within 30 d	66.4 (2878)	62.6 (4285)	70.2 (3784)	66.6 (5413)	78.0 (2850)	75.3 (4774)
Women	35.6 (1543)	37.8 (2585)	43.9 (2367)	46.9 (3815)	55.5 (2027)	62.1 (3939)
Mean age (SD)	65.9 (2.7)	65.9 (2.7)	74.9 (2.8)	74.7 (2.9)	85.1 (3.8)	86.1 (4.4)
Comorbidity						
Mean CHA_2_DS_2_‐VASc score (SD)	2.2 (1.2)	2.1 (1.2)	3.3 (1.3)	3.1 (1.2)	4.0 (1.2)	3.9 (1.1)
Mean HAS‐BLED score (SD)	2.0 (1.1)	1.9 (1.0)	2.5 (0.9)	2.4 (0.9)	2.6 (0.9)	2.4 (0.9)
Mean Charlson Comorbidity Index score (SD)	0.9 (1.4)	0.8 (1.4)	1.2 (1.6)	1.1 (1.6)	1.2 (1.6)	1.3 (1.6)
Renal dysfunction	6.5 (280)	2.8 (189)	8.7 (471)	4.3 (351)	10.0 (366)	5.1 (323)
Myocardial infarction	11.0 (475)	8.0 (550)	15.0 (809)	10.2 (829)	17.1 (625)	13.0 (825)
Heart failure	16.9 (731)	11.2 (765)	21.1 (1136)	15.5 (1263)	34.2 (1250)	28.0 (1774)
Peripheral arterial disease	6.1 (265)	5.0 (341)	10.4 (562)	7.5 (608)	10.5 (383)	8.8 (559)
Vascular disease	15.7 (679)	12.1 (830)	22.4 (1204)	16.2 (1318)	24.9 (908)	19.8 (1254)
Diabetes mellitus	14.2 (615)	11.1 (761)	13.6 (731)	11.6 (940)	11.6 (422)	10.8 (686)
Hyperlipidemia	15.7 (678)	12.8 (877)	17.4 (937)	14.3 (1164)	13.1 (478)	10.6 (672)
Hypertension	60.7 (2629)	58.3 (3992)	65.0 (3504)	62.2 (5057)	69.1 (2525)	65.0 (4119)
Cancer	4.6 (198)	3.6 (246)	6.8 (365)	5.8 (472)	6.3 (229)	6.1 (385)
Chronic pulmonary disease	12.2 (530)	11.1 (760)	17.2 (926)	15.8 (1280)	17.1 (625)	17.4 (1103)
Prior bleeding	10.3 (446)	9.5 (651)	13.0 (700)	13.1 (1063)	15.6 (569)	14.7 (931)
Depression	1.4 (60)	1.3 (91)	1.5 (79)	1.7 (140)	2.2 (80)	2.9 (183)
Hospital‐diagnosed obesity	10.2 (440)	9.6 (657)	8.0 (429)	7.8 (634)	4.8 (177)	4.8 (305)
Smoking‐related diagnoses	4.4 (192)	4.7 (323)	4.7 (254)	5.0 (404)	2.7 (99)	3.7 (236)
Alcohol‐related abuse	4.3 (186)	4.6 (314)	2.5 (132)	3.2 (258)	1.1 (42)	1.2 (78)
Drug‐related abuse	0.2 (7)	0.2 (11)	0.1 (8)	0.2 (15)	0.1 (5)	0.2 (12)
Comedications						
Clopidogrel	3.8 (164)	2.5 (173)	4.5 (241)	3.7 (298)	4.6 (167)	4.5 (284)
Aspirin	39.2 (1696)	34.1 (2337)	45.8 (2466)	38.0 (3085)	49.8 (1821)	44.5 (2821)
Loop diuretics	16.9 (734)	11.2 (768)	21.2 (1143)	15.7 (1272)	34.2 (1250)	28.1 (1779)
Nonloop diuretics	33.0 (1428)	32.6 (2233)	39.7 (2137)	38.1 (3093)	45.4 (1658)	42.1 (2669)
Digoxin	5.5 (238)	3.6 (248)	6.6 (358)	4.8 (387)	9.2 (337)	8.5 (541)
β‐Blockers	47.0 (2035)	38.8 (2656)	44.2 (2380)	37.9 (3078)	45.3 (1655)	37.8 (2395)
Calcium channel blockers	29.4 (1275)	27.0 (1848)	32.5 (1751)	31.1 (2529)	37.2 (1358)	32.7 (2075)
Verapamil	4.0 (172)	3.1 (212)	3.0 (162)	2.3 (186)	2.4 (86)	2.4 (153)
Renin‐angiotensin inhibitors	45.2 (1960)	44.3 (3034)	47.9 (2578)	47.2 (3839)	50.5 (1843)	46.0 (2915)
Statin	37.4 (1619)	35.3 (2415)	43.3 (2333)	38.6 (3139)	34.4 (1256)	30.3 (1921)
NSAIDs	23.3 (1008)	23.9 (1639)	23.1 (1242)	23.3 (1895)	18.9 (691)	18.4 (1169)

AF indicates atrial fibrillation; IQR, interquartile range; NOAC, nonvitamin K antagonist oral anticoagulant; NSAIDs, nonsteroidal anti‐inflammatory drugs; OAC, oral anticoagulant.

### 180‐Day Landmark Populations

A total of 956 patients died before the date of the 180‐day landmark and 110 were diagnosed with dementia, leaving 33 617 patients in the 180‐day landmark population (Figure 1). Table S4 and Figure S1 illustrates the change in patient characteristics from index OAC prescription to the 180‐day landmark. The prevalence of most hospital‐diagnosed comorbidity changed little, with slight increases in mean risk and comorbidity scores. However, diagnoses of heart failure increased markedly over the landmark period. Comedication use also increased, in particular cardiovascular medicine such as loop diuretics, digoxin, and β‐blockers.

After weighing the study populations using the inverse probability of treatment weighting method, most baseline standardized differences were below the 0.1 threshold, indicating that the weighted treatment regimens were comparable within each age group with regard to measured covariates (Figure S2). Inspection of individual propensity score distributions showed sufficient overlap between treatment populations in order to obtain a valid comparison (Figure S3).

### Dementia Risk

**Figure 2:**
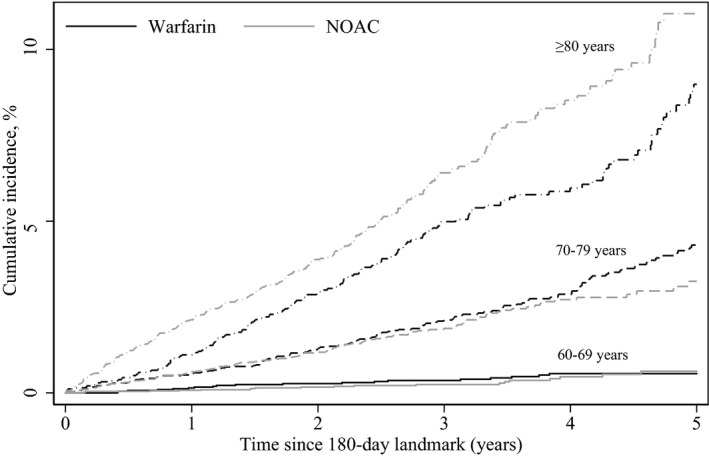
Propensity‐weighted cumulative risk of dementia by age and treatment regimen since the 180‐day landmark. NOAC indicates nonvitamin K antagonist oral anticoagulant.

The mean follow‐up was 3.4 years (SD 1.6); 3.7 for patients aged 60 to 69 years (3.4 years for NOACs and 4.3 years for warfarin), 3.4 for patients aged 70 to 79 years (3.1 years for NOACs and 3.9 years for warfarin), and 2.9 for patients 80 years and older (2.7 years for NOACs and 3.3 years for warfarin). Figure 2 displays cumulative incidence of dementia by treatment regimen and age. During follow‐up, 41 patients aged 60 to 69 years developed dementia, 276 patients aged 70 to 79 years developed dementia, and 441 patients 80 years and older developed dementia (Table 2).

**Table 2:** Number of Events and Crude and Weighted Rates of Dementia Per 100 Person‐Years by Age and Treatment Regimen

Outcome	Age 60 to 69 y		Age 70 to 79 y		Age 80 y and Older	
	NOAC	Warfarin	NOAC	Warfarin	NOAC	Warfarin
No. of events	20	21	133	143	279	162
Crude rate	0.11	0.12	0.64	0.80	2.16	1.65
Weighted rate	0.11	0.12	0.64	0.78	2.16	1.70

NOAC indicates nonvitamin K antagonist oral anticoagulant.

**Figure 3:**
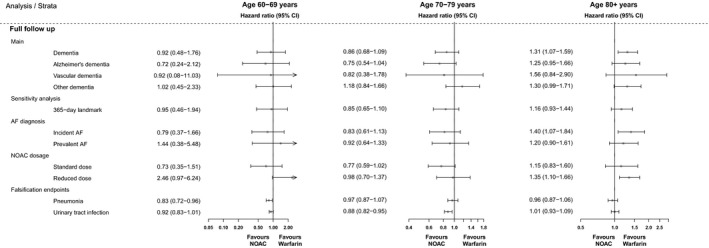
Forest plots of propensity‐weighted study outcomes by age and treatment regimen. AF indicates atrial fibrillation; NOAC, nonvitamin K antagonist oral anticoagulant.

Among patients aged 60 to 69 years, rates of dementia were nonsignificantly lower between NOAC versus warfarin users (0.11 per 100 person‐years versus 0.12); weighted HR at full follow‐up, 0.92 (95% CI, 0.48–1.76) (Table 2, Figure 3). For patients aged 70 to 79 years, NOAC use was also associated with lower rates of dementia compared with warfarin use (0.64 per 100 person‐years versus 0.78 per 100 person‐years; weighted HR, 0.86 [95% CI, 0.89–1.09]), whereas NOAC use was associated with increased rates of dementia compared with warfarin use (2.16 per 100 person‐years versus 1.70 per 100 person‐years, weighted HR, 1.31 [95%, 1.07–1.59]) in patients 80 years and older (Figure 3).

Similar patterns were also evident for the specific dementia subtypes, although there were few events yielding imprecise estimates with wide CIs (Figure 3), and when using a 365‐landmark, stratifying by incident versus nonincident AF, or restricting the analyses alternately to patients with standard or reduced doses of NOACs.

### Falsification End Points

Analyses of the treatment‐associated falsification end points yielded weighted hazard rates around the null for most comparisons, indicating that the a priori hypothesis of neutral associations were fulfilled (Figure 3, Table S5).

## Discussion

In this nationwide cohort study investigating OAC‐naive patients with nonvalvular AF and no prior neurological diagnoses, we observed nonsignificantly lower rates of dementia among NOAC users younger than 80 years relative to warfarin users, whereas rates were higher among NOAC users in patients 80 years and older.

### Comparison With Previous Studies

In the absence of disease‐modifying treatments for most forms of dementia, any means to prevent or delay its onset is of major clinical and social importance.^34^ Accumulating evidence suggests an association between AF and dementia via various pathways including occurrence of overt or silent strokes, lower cardiac output in AF leading to cerebral hypoperfusion, and/or presence of shared risk factors for AF and dementia.^4^, ^5^, ^6^, ^7^, ^8^, ^35^

Nevertheless, it is less clear to what extent dementia development in AF is affected by OAC treatment. Prior studies have indicated that the quality of management of warfarin therapy as measured by TTR is associated with risk of dementia.^6^, ^11^ The hypothesis being that periods of supratherapeutic or subtherapeutic anticoagulation predispose patients to microbleeds and microthrombi, which may eventually lead to dementia; hence, dementia could be considered a potential long‐term complication of warfarin therapy. This line of thinking represents a challenging clinical situation with a therapy used to prevent life‐threatening strokes that potentially lead to long‐term cognitive defects. On the other hand, a recent Swedish cohort study found a 29% lower relative risk of dementia in patients with AF treated with OACs compared with patients with AF without OAC treatment,^13^ suggesting that it is likely not anticoagulation per se but the extent of exposure to overcoagulation and/or undercoagulation that could confer an increased risk of dementia.

A central question is whether optimized treatment of AF can prevent or delay the onset of dementia. It has been hypothesized that NOACs could play a role in reducing the risk of cognitive impairment and dementia in patients with AF but the available evidence is sparse and equivocal.^13^, ^14^ In a US cohort of patients receiving long‐term anticoagulation with either NOACs or warfarin, Jacobs et al^14^ reported a lower rate of dementia in NOAC users (0.3%. versus 0.7%).^14^ However, the duration of follow‐up was short (median 309 days for warfarin users versus 185 days for NOAC users), and the study population included patients with prior OAC use as well as stroke. Using data from 2 large US claims databases, Chen et al^36^ recently compared the incidence of dementia in patients with AF initiating treatment with different OACs. Based on 6 sets of 1:1 pairwise propensity score–matched comparisons of different OAC regimens, the authors concluded that NOACs were associated with a lower incidence of dementia compared with warfarin, with HRs ranging from 0.73 to 0.79 according to choice of NOAC. In line with our findings, Friberg et al^13^ found comparable rates of dementia in Sweden when comparing propensity‐matched cohorts of warfarin users and NOAC users (HR, 0.97; 95% CI, 0.67–1.40).

By including a large nationwide cohort of OAC‐naive patients without previous neurological diagnoses, our study extends these previous studies. Our finding of comparable rates of dementia across treatment regimens may potentially not be generalizable to other populations given the presumably better quality of anticoagulation control with warfarin in both the Danish and the Swedish settings with TTR >70% in routine clinical care^37^, ^38^ compared with (for example) a TTR of 54% among 138 319 patients treated in US physician practices.^39^ A randomized trial comparing the incidence of dementia in patients with AF randomized to either warfarin or dabigatran was initiated in 2017 with estimated completion in 2021 (ClinicalTrials.gov identifier: NCT03061006).

### Study Strengths

The registry databases facilitated our implementation of a large well‐defined, nationwide, population‐based cohort of patients with nonvalvular AF initiating OAC treatment during 2011 through 2016. The tax‐supported healthcare system for the entire Danish population includes free access to medical care and partial reimbursement of prescribed medications,^15^ leading to minimal disparity in access to healthcare services in this study. Also, analyses of the treatment‐associated falsification end points found that the a priori hypothesis of neutral associations was fulfilled.

## Study Limitations

There are also limitations to our study. Most importantly, data were drawn from administrative registries. Because of the observational nature of our study, residual or unmeasured confounding is likely to persist. For instance, patients with cognitive impairment have been shown to have poorer control of their international normalized ratio,^40^ and we cannot exclude that the increased risk of dementia associated with NOACs in patients 80 years and older could represent channeling of NOACs toward patients with prodromal or undiagnosed prevalent dementia. We did not have access to information on treatment adherence and TTR among warfarin users, nor did we have clinical data on the severity and control of comorbidities or laboratory (eg, renal function), anthropometric, or socioeconomic data, which could have improved our ability to control confounding. Another limitation is the relatively short duration of follow‐up when considering the induction period for dementia development, and our follow‐up period (mean 3.4 years) may not have been long enough to detect a differential effect of treatment regimen on dementia development. Furthermore, we lacked results of diagnostic brain imaging and objective measures of cognitive function. The diagnosis of dementia has a high positive predictive value, whereas the sensitivity is unknown.^22^

## Conclusions

In this propensity‐weighted Danish nationwide cohort of OAC‐naive patients with AF with no prior neurological diagnoses, there was no clinically meaningful difference in dementia development between the majority of users of NOAC or warfarin, apart from those 80 years and older, where a higher risk was noted in NOAC users. Potential residual confounding related to selective prescribing and unobserved comorbidities warrant cautious clinical interpretation of our findings.

## Sources of Funding

The Obel Family Foundation partly funded this research by an unrestricted grant. The sponsor had no role in the design and conduct of the study; collection, management, analysis, and interpretation of the data; writing of the report; or the decision to submit the article for publication.

## Disclosures

All authors have completed the ICMJE Form for Disclosure of Potential Conflicts of Interest. Dr Lip has received consulting fees from Bayer/Janssen, BMS/Pfizer, Biotronik, Medtronic, Boehringer Ingelheim, Microlife, and Daiichi‐Sankyo; and is a speaker for Bayer, BMS/Pfizer, Medtronic, Boehringer Ingelheim, Microlife, Roche, and Daiichi‐Sankyo. Dr Larsen has served as an investigator for Janssen Scientific Affairs, LLC, and Boehringer Ingelheim and received speaking fees from Bayer, BMS/Pfizer, Boehringer Ingelheim, MSD, and AstraZeneca. Mr Nielsen has received speaking fees from Boehringer Ingelheim, consulting fees from Bayer, and grant support from BMS/Pfizer. Mr Skjøth has received consulting fees from Bayer. The remaining authors have no disclosures to report.

## Supporting information


**Table S1.** Definitions on Comorbidity and Concomitant Medication According to *ICD‐10* Codes and ATC Codes
**Table S2**. Risk Score Definitions
**Table S**3. Definition of Charlson Comorbidity Index
**Table S4**. Patient Characteristics by Age at Time of Index Oral Anticoagulant Prescription Claim and at the 180‐Day Landmark Before Propensity Weighting
**Table S5.** Crude and Weighed HRs for the Association Between Exposure and Falsification End Points
**Figure S1**. Change in patient characteristics by age from the time of index oral anticoagulant prescription claim (day 0) to the 180‐day landmark before propensity weighting visualized by signed standardized differences
**Figure S2.** Plot of maximum of pairwise standardized differences for patient characteristics of nonvitamin K antagonist oral anticoagulant (NOAC) users and warfarin users at the 180‐day landmark stratified by age before and after applying propensity (inverse probability of treatment) weighting
**Figure S3.** Propensity score distributions for warfarin and nonvitamin K antagonist oral anticoagulants (NOACs) by age group.
