# Supplementary material for: Nonvitamin K Antagonist Oral Anticoagulants Versus Warfarin in Atrial Fibrillation Patients and Risk of Dementia: A Nationwide Propensity‐Weighted Cohort Study
Source: J Am Heart Assoc. 2019 May 29;8(11):e011358. doi: 10.1161/JAHA.118.011358 (PMC6585353; doi:10.1161/JAHA.118.011358)

SUPPLEMENTAL MATERIAL

Table S1. Definitions on comorbidity and concomitant medication according to ICD-10 codes and ATC-codes.

Disease	ICD-8 codes	ICD-10 codes	ATC-code
**Study population**			
AF	42793 42794	I48	
**Exposure**			
Warfarin			B01AA03
Dabigatran			B01AE07
Rivaroxaban			B01AF01
Apixaban			B01AF02
**Outcome**			
Dementia			
Alzheimer's disease	290.10, 290.09	F00, G30	
Vascular dementia	293.09, 293.19	F01	
Other dementia	094.19, 290.11-290.19	F02-F03, F10.73-F19.73, G23.1, G31.0A, G31.0B, G31.1, G31.8B, G31.8E, G31.85	
**Exclusion criteria**			
PE and/or DVT within 90 days before index date			
Valvular AF			
Mitral stenosis		I05	
Mechanical heart valve		Z952 Z953 Z954	
Ischemic stroke	433-434	I63	
Hemorrhagic stroke/intracerebral bleeding	430 431	I60 I61 I62	
Unspecified stroke	436	I64	
Transient ischemic disease	43509 43599	G45	
other cerebrovascular disease	432, 437, 438	I62, 165-169, G46	
Traumatic intracranial bleeding		S063C S064 S065 S066	
Hemiplegia	344	G81, G82	
Dementia	094.19, 290.09-290.19, 292.09, 293.09, 293.19	F00-F03, G30, F1x.73 series (F10.73, F1173, F1273, F1373, F1473, F1573, F1673, F1873-F19.73), G23.1, G31.0, G31.0A, G31.0B, G31.1, G31.8B, G31.8E, G31.85	
Cognitive impairment	-	F06	
Amnestic syndromes	291.19	F04, F04.9, F05.1, F10.6, F18.6, F19.6	
Head trauma	850.99, 851.29-854.99, 800.99-801.09, 803.99	S06.0, S06.1-S06.9, S02.0-S02.1, S02.7, S02.9	

**Comorbidity (shared risk factors)**			
**Underlying vascular diseases**			
Myocardial infarction	410	I21 I23	
Heart failure	42709 42710 42711 42719 42899 78249	I110 I130 I132 I50	
Peripheral arterial disease	440 441 442 443 444 445	I702 I703 I704 I705 I706 I707 I708 I709 I71 I739 I74	
Diabetes	24900 24909 25008 25009	E10 E11	A10
Hyperlipidemia / hypercholesterolemia	272.00-272.09<br>279.00<br>279.01	E780-E782<br>E784<br>E785	
Hypertension			See specified definition below[a]
**Other comorbidities**			
Cancer		C	
Renal disease			
Bleeding			
**Smoking-related diagnoses**			
Chronic pulmonary disease	490-493, 515-518	J40-J47, J60-J67, J68.4, J70.1	
Diagnosis of problems related to active smoking	-	Z720A, Z720E	
Smoking prevention counselling or smoking cessation intervention	-	Treatment codes: BQFT01, BQFS01	
Current or former smoking status	-	Administrative procedure codes: ZZP01A1A, ZZP01A1B2, ZZP0020	
**Hospital diagnosed obesity**	277	E65-E68	
**Depression**	296.09, 296.29, 296.99, 298.09, 300.49, 300.19	F32, F33	
**Substance abuse diagnoses and related comorbidities**			
**Drug-related abuse**			
Opioids	304.09, 304.19	F11.0-F11.9	
Cannabis	304.59	F12.0-F12.9	
Sedatives/hypnotics	304.29, 304.39	F13.0-F13.9	
Cocaine	304.49	F14.0-F14.9	
Other stimulants	304.69	F15.0-F15.9	
Hallucinogens	304.79	F16.0-F16.9	
Other and multiple drugs	304.89, 304.99	F18.0-F19.9	
**Alcohol-related abuse**			

Alcohol psychosis, and alcohol abuse syndrome	291.09-291.99, 303.09-303.99	F10.2-F10.9	
Alcohol related diseases	571.09, 571.10, 577.10, 979.59	G31.2, G62.1, G72.1, I42.6, K29.2, K70, K86.0, T50.0A, Z72.1	
**Co-medications**			
Diabetes mellitus			A10
Clopidogrel			B01AC04
Aspirin			B01AC06
Thienopyridines (clopidogel, tricagrelor, prasugrel)			B01AC04 B01AC24 B01AC22
Loop diuretics			C03C
Digoxin			C01AA05
Dronedarone			C01BD07
Hypertension			C02
Non-loop diuretics			C02DA C02L C03A C03B C03D C03E C03X C07C C07D C08G C09BA C09DA C09XA52
Congestive heart failure			C03C
Beta blocker			C07
Calcium channel blocker			C07F C08 C09BB C09DB
Verapamil			C08DA01
Renin-angiotensin inhibitor (ARB or ACE inhibitor)			C09
Statins			C10
NSAIDs			M01A
**Dementia medications**			
Anti-dementia medications			N06D
Donepezil			N06DA02
Rivastigmin			N06DA03
Galantamin			N06DA04
Memantin			N06DX01

[a]We identified subjects with hypertension from combination treatment with at least two of the following classes of antihypertensive drugs:
I· Alpha adrenergic blockers (C02A, C02B, C02C)
II· Non-loop diuretics (C02DA, C02L, C03A, C03B, C03D, C03E, C03X, C07C, C07D, C08G, C09BA, C09DA, C09XA52)
III· Vasodilators (C02DB, C02DD, C02DG, C04, C05)
IV· Beta blockers (C07)
V· Calcium channel blockers (C07F, C08, C09BB, C09DB)
VI· Renin-angiotensin system inhibitors (C09)

Table S2. Risk score definitions.

Risk score	Points if present
CHA_2DS_2VASc[*]	
Congestive heart failure or Left Ventricular Dysfunction	1
Hypertension	1
Age ≥ 65 years	1
Age ≥ 75 years	1
Diabetes mellitus	1
Stroke (ischemic stroke, transient ischemic disease or systemic embolism)	2
Vascular disease (myocardial infarction, peripheral arterial disease, or aortic plaque)	1
Sex category (female)	1
HAS-BLED[†]	
Hypertension	1
Abnormal renal function	1
Abnormal hepatic function	1
Stroke (ischemic stroke or transient ischemic attack)	1
Bleeding	1
Labile international normalized ratio[£]	1
Elderly age (≥ 65 years)	1
Drugs (aspirin, clopidogrel, or non-steroidal anti-inflammatory drugs)	1
Alcohol intake	1

[*]Reflects stroke risk in atrial fibrillation patients not in anticoagulant therapy (Lip GYH, Nieuwlaat R, Pisters R, Lane DA, Crijns HJGM. Refining clinical risk stratification for predicting stroke and thromboembolism in atrial fibrillation using a novel risk factor-based approach: the euro heart survey on atrial fibrillation. (Chest 2010;137:263-72)

[†]Reflects bleeding risk in atrial fibrillation patients undergoing anticoagulant therapy (Pisters R, Lane DA, Nieuwlaat R, de Vos CB, Crijns HJGM, Lip GYH. A novel user-friendly score (HAS-BLED) to assess 1-year risk of major bleeding in patients with atrial fibrillation: the Euro Heart Survey. Chest 2010;138:1093-100).

[£]Not included due to unavailable information

Table S3. Definition of Charlson Comorbidity Index.

Weight	Condition	ICD-10	ICD-8
1	Myocardial infarction	I21 I22 I23	410
1	Congestive heart failure	I50 I110 I130 I132	42709 42710 42711 42719 42899 78249
1	Peripheral vascular disease	I70 I71 I72 I73 I74 I77	440 441 442 443 444 445
1	Cerebrovascular disease	I60 I61 I62 I63 I64 I65 I66 I67 I68 I69 G45 G46	430 431 432 433 434 435 436 437 438
1	Dementia	F00 F01 F02 F03 F051 G30	29009 29010 29011 29012 29013 29014 29015 29016 29017 29018 29019 29309
1	Cronic pulmonary disease	J40 J41 J42 J43 J44 J45 J46 J47 J60 J61 J62 J63 J64 J65 J66 J67 J684 J701 J703 J841 J920 J961 J982 J983	490 491 492 493 515 516 517 518
1	Connective tissue disease	M05 M06 M08 M09 M30 M31 M32 M33 M34 M35 M36 D86	712 716 734 446 13599
1	Ulcer disease	K221 K25 K26 K27 K28	53091 53098 531 532 533 534
1	Mild lever disease	B18 K700 K701 K702 K703 K709 K71 K73 K74 K760	571 57301 57304
1	Diabetes Mellitus	E10 E11 E12 E13 E14 O240 O241 O242 O243 O245 O246 O247 O248 O249 H360	249 250
2	Hemiplegia	G81 G82	344
2	Moderate to severe renal disease	I12 I13 N00 N01 N02 N03 N04 N05 N07 N11 N14 N17 N18 N19 Q61	403 404 580 581 582 583 584 59009 59319 7531 792

2	Diabetes with chronic complications	E102 E103 E104 E105 E106 E107 E108 E112 E113 E114 E115 E116 E117 E118	24901 24902 24903 24904 24905 24908 25001 25002 25003 25004 25005 25008
2	Any tumor	C0 C1 C2 C3 C4 C5 C6 C70 C71 C72 C73 C74 C75	14 15 16 17 18 190 191 192 193194
2	Leukemia	C91 C92 C93 C94 C95	204 205 206 207
2	Lymphoma	C81 C82 C83 C84 C85 C88 C90 C96	200 201 202 203 27559
3	Moderate to severe liver disease	B150 B160 B162 B190 K704 K72 K766 I85	07000 07002 07004 07006 07008 57300 4560
6	Metastatic solid tumor	C76 C77 C78 C79 C80	195 196 197 198 199
6	AIDS	B21 B22 B23 B24	07983

Charlson ME, Pompei P, Ales KL, MacKenzie CR. A new method of classifying prognostic comorbidity in longitudinal studies: development and validation. J Chronic Dis 1987; 40: 373–383.

Table S4. Patient characteristics by age at time of index oral anticoagulant prescription claim and at the 180-day landmark before propensity-weighting.

	60-69 years		70-79 years		80 years and older	
Characteristic, % (N)	Index	180-day landmark	Index	180-day landmark	Index	180-day landmark
N	11052	11052	13237	13237	9328	9328
Female sex	37.0 (4085)	37.0 (4085)	45.7 (6046)	45.7 (6046)	59.8 (5581)	59.8 (5581)
Mean age (sd)*	65.9 (2.7)	65.9 (2.7)	74.8 (2.9)	74.8 (2.9)	85.6 (4.1)	85.6 (4.1)
Mean CHA_2DS_2-VASc score (sd)	2.1 (1.2)	2.3 (1.2)	3.1 (1.3)	3.3 (1.3)	4.0 (1.1)	4.1 (1.2)
Mean HAS-BLED score (sd)	1.9 (1.0)	2.1 (1.0)	2.4 (0.9)	2.5 (0.9)	2.5 (0.9)	2.5 (0.9)
Mean Charlson Comorbidity score (sd)	0.8 (1.4)	0.8 (1.4)	1.1 (1.5)	1.1 (1.5)	1.2 (1.6)	1.2 (1.6)
Myocardial infarction*	9.1 (1011)	9.5 (1047)	12.0 (1587)	12.4 (1648)	14.2 (1320)	14.7 (1367)
Heart failure*	13.0 (1440)	26.0 (2868)	17.2 (2276)	33.1 (4382)	29.0 (2708)	50.5 (4709)
Peripheral arterial disease*	5.3 (586)	6.0 (659)	8.5 (1120)	9.1 (1210)	9.0 (839)	9.8 (912)
Vascular disease*	13.4 (1478)	14.2 (1570)	18.4 (2436)	19.4 (2564)	20.9 (1953)	22.0 (2051)
Diabetes*	12.1 (1342)	13.4 (1478)	12.1 (1600)	13.2 (1745)	10.9 (1013)	11.5 (1069)
Hyperlipdemia*	13.9 (1534)	15.8 (1744)	15.4 (2042)	16.8 (2228)	11.5 (1075)	12.3 (1151)
Hypertension*	59.1 (6533)	66.2 (7312)	63.2 (8366)	68.6 (9074)	66.5 (6207)	70.6 (6585)
Cancer	3.7 (412)	5.2 (576)	5.8 (773)	8.0 (1053)	5.9 (548)	8.5 (794)
Chronic pulmonary disease	11.2 (1240)	12.5 (1381)	16.0 (2119)	17.6 (2326)	16.4 (1534)	17.9 (1670)
Liver disease	0.4 (40)	0.4 (45)	0.3 (36)	0.3 (41)	0.2 (17)	0.2 (19)
Renal dysfunction	4.1 (453)	4.8 (532)	5.8 (774)	7.0 (921)	6.7 (622)	8.1 (755)
Prior bleeding	9.6 (1065)	10.9 (1201)	12.9 (1709)	14.5 (1920)	14.9 (1393)	16.7 (1558)
Depression	1.3 (144)	1.4 (156)	1.5 (205)	1.7 (225)	2.5 (235)	3.0 (284)
Hospital diagnosed obesity	9.8 (1078)	10.8 (1195)	7.8 (1030)	8.3 (1095)	4.8 (444)	4.9 (460)
Smoking-related diagnoses*	4.4 (486)	5.3 (587)	4.7 (620)	6.1 (811)	3.1 (293)	4.3 (399)
Alcohol-related abuse*	4.4 (483)	4.6 (505)	2.8 (372)	3.0 (391)	1.1 (104)	1.2 (112)

Drug-related abuse*	0.2 (17)	0.2 (17)	0.2 (22)	0.2 (23)	0.2 (14)	0.2 (14)
Clopidogrel	3.0 (330)	5.0 (554)	3.9 (522)	6.7 (881)	4.6 (426)	7.0 (657)
Aspirin	36.0 (3981)	37.4 (4132)	40.9 (5413)	41.8 (5534)	46.2 (4314)	46.0 (4294)
Loop diuretics	13.1 (1446)	26.0 (2873)	17.3 (2292)	33.2 (4394)	29.1 (2713)	50.5 (4713)
Non-loop diuretics	32.7 (3612)	39.4 (4358)	38.7 (5117)	43.4 (5742)	43.2 (4028)	47.4 (4417)
Digoxin	4.2 (464)	22.1 (2443)	5.3 (708)	25.3 (3354)	8.6 (798)	34.0 (3171)
Beta blocker	41.9 (4630)	82.4 (9111)	40.2 (5322)	80.0 (10586)	40.7 (3792)	75.3 (7024)
Calcium channel blocker	27.9 (3089)	32.4 (3580)	31.6 (4185)	35.0 (4627)	34.5 (3214)	36.3 (3390)
Verapamil	3.4 (379)	6.6 (729)	2.6 (341)	5.0 (663)	2.4 (221)	3.7 (344)
Renin-angiotensin inhibitor	44.6 (4933)	54.8 (6055)	47.4 (6269)	55.8 (7385)	47.8 (4455)	54.4 (5070)
Statins	36.0 (3984)	42.8 (4734)	40.5 (5363)	45.4 (6009)	32.0 (2986)	34.5 (3222)

*Included in the propensity score

Table S5. Crude and weighed hazard ratios for the association between exposure and falsification endpoints.

**Falsification endpoint**	**60-69 years**<br>**Hazard ratio (95% CI)**	**70-79 years**<br>**Hazard ratio (95% CI)**	**80 years and older**<br>**Hazard ratio (95% CI)**
**Pneumonia**			
Crude analysis	0.79 (0.69-0.91)	0.91 (0.82-1.01)	0.97 (0.88-1.06)
Weighted analysis	0.83 (0.72− 0.96)	0.97 (0.87− 1.07)	0.96 (0.87− 1.06)
**Urinary tract infection**			
Crude analysis	0.92 (0.84-1.01)	0.89 (0.82-0.96)	1.07 (0.99-1.16)
Weighted analysis	0.92 (0.83− 1.01)	0.88 (0.82− 0.95)	1.01 (0.93− 1.09)

Figure S1. Change in patient characteristics by age from the time of index oral anticoagulant prescription claim (day 0) to the 180-day landmark before propensity-weighting visualized by signed standardized differences.

Patients aged 60-69 years

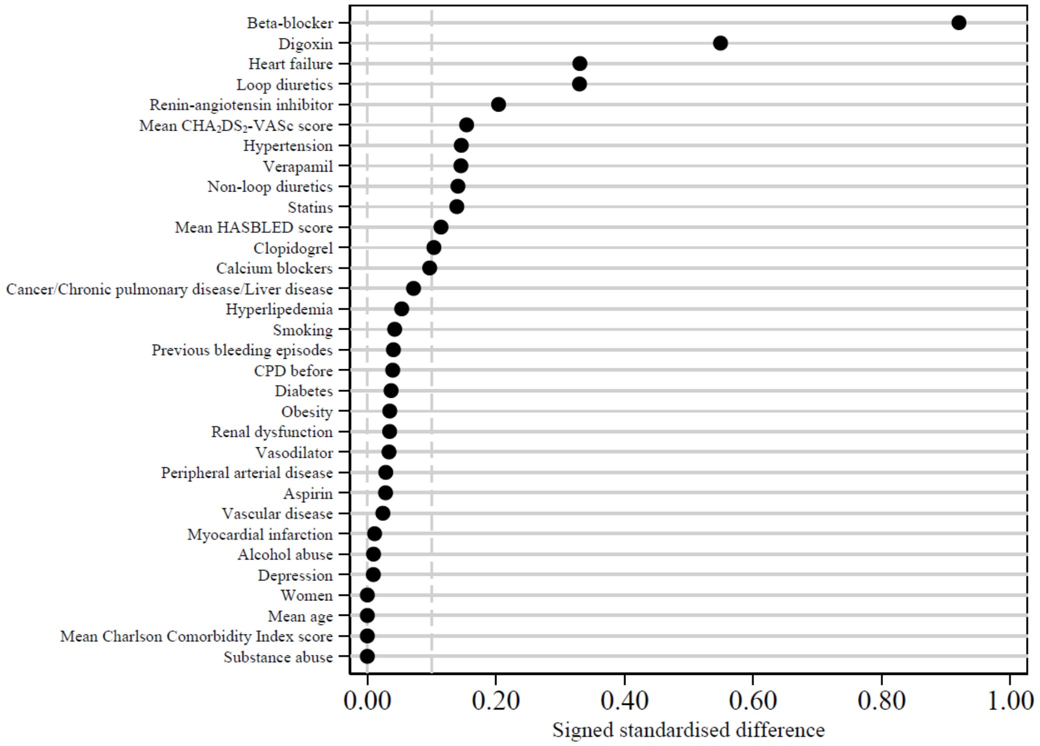

Patients aged 70-79 years

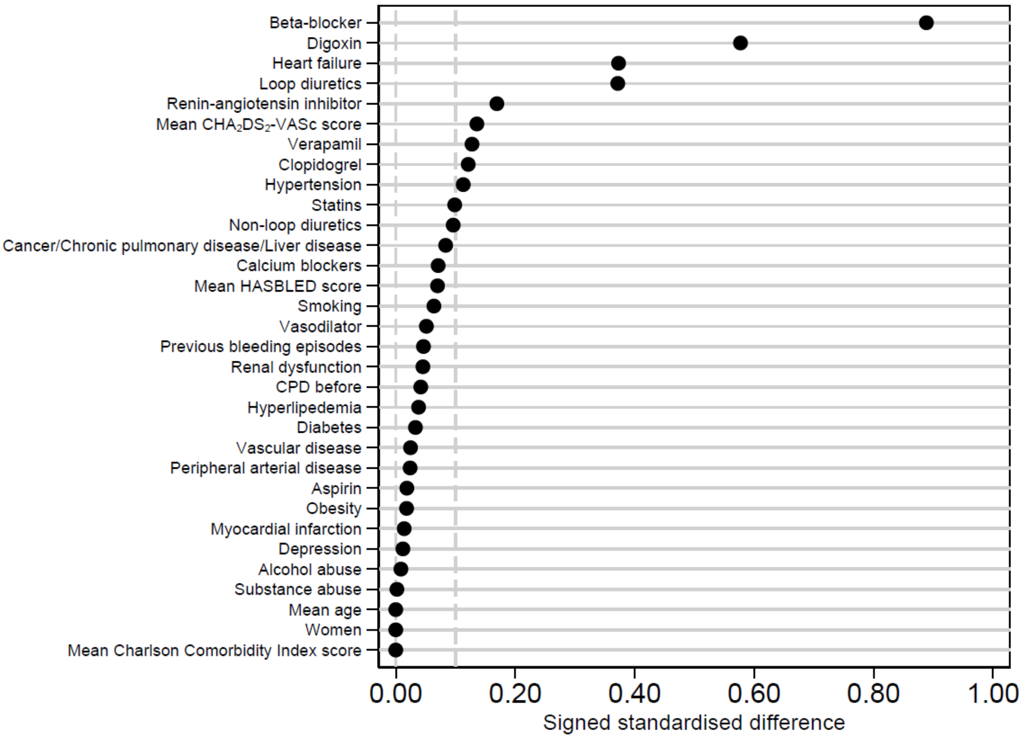

Patients aged 80 years and older

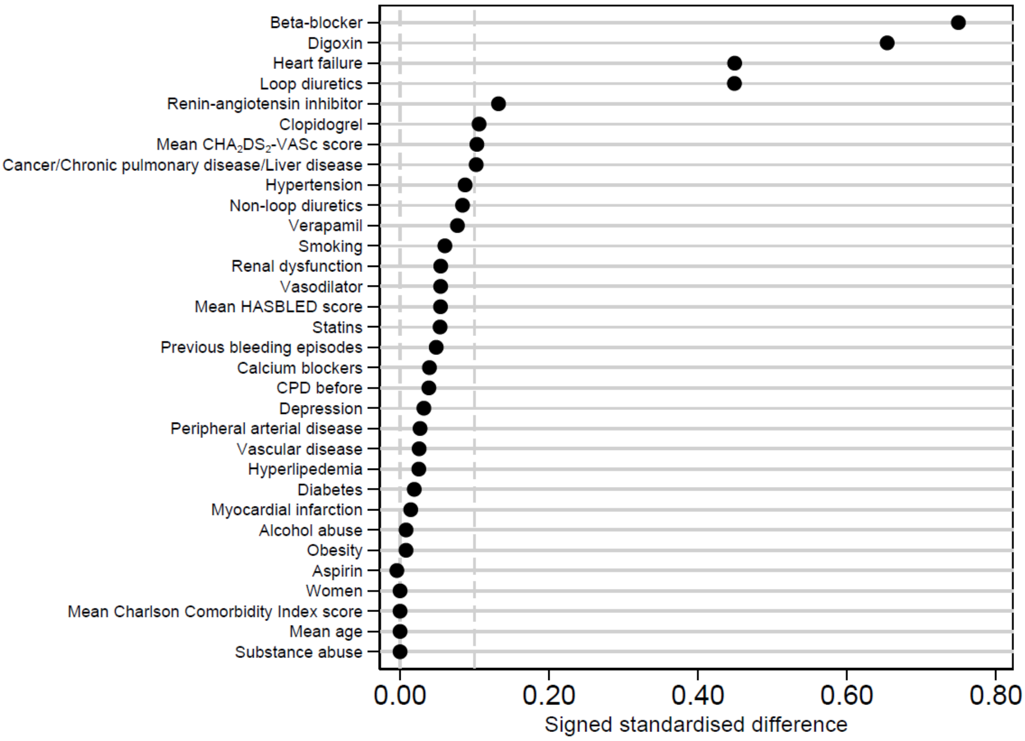

Figure S2. Plot of maximum of pairwise standardized differences for patient characteristics of NOAC users and warfarin users at the 180-day landmark stratified by age before and after applying propensity weights (IPTW).

Patients aged 60-69 years

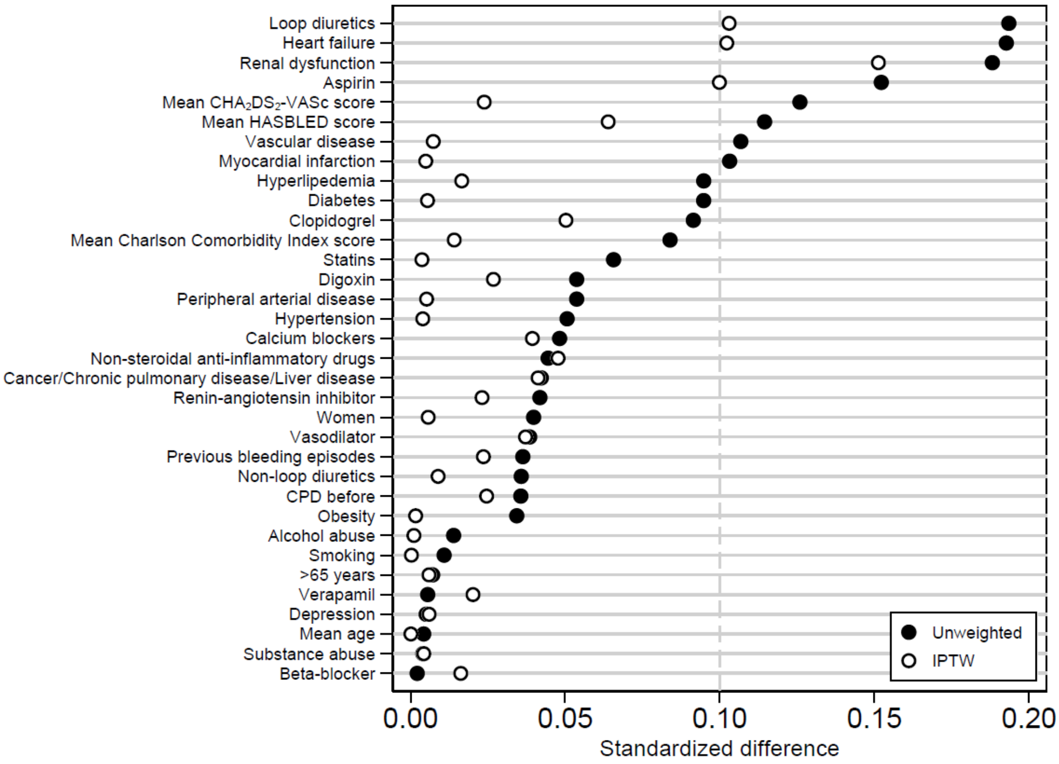

Patients aged 70-79 years

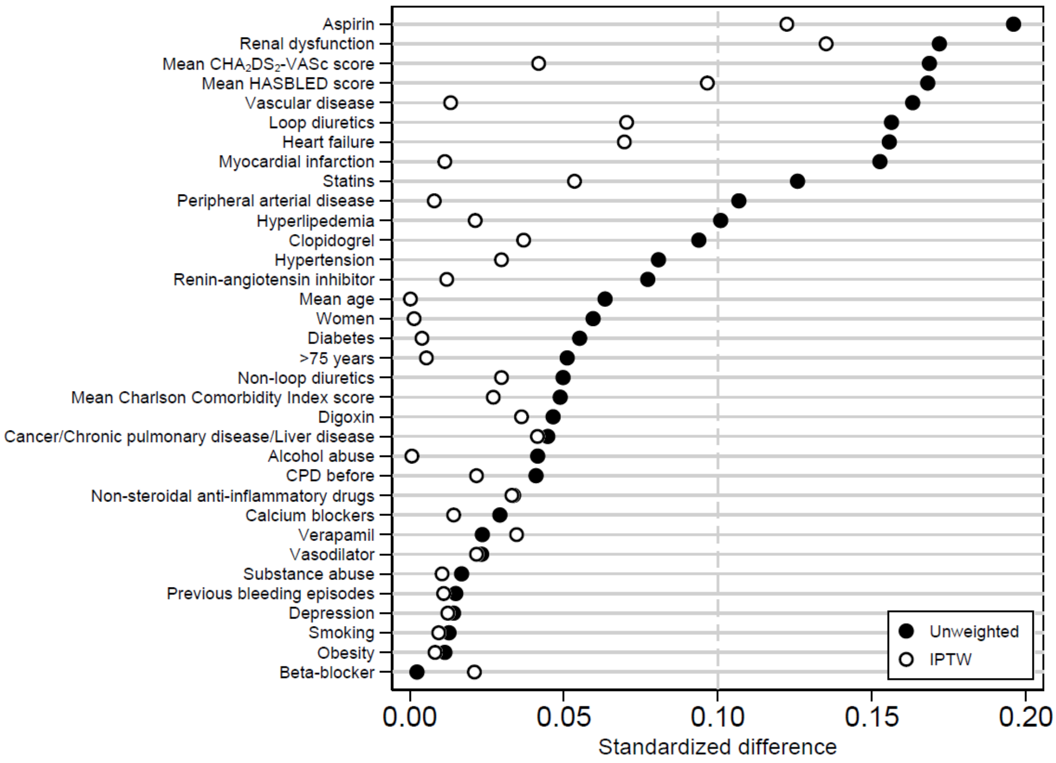

Patients aged 80 years and older

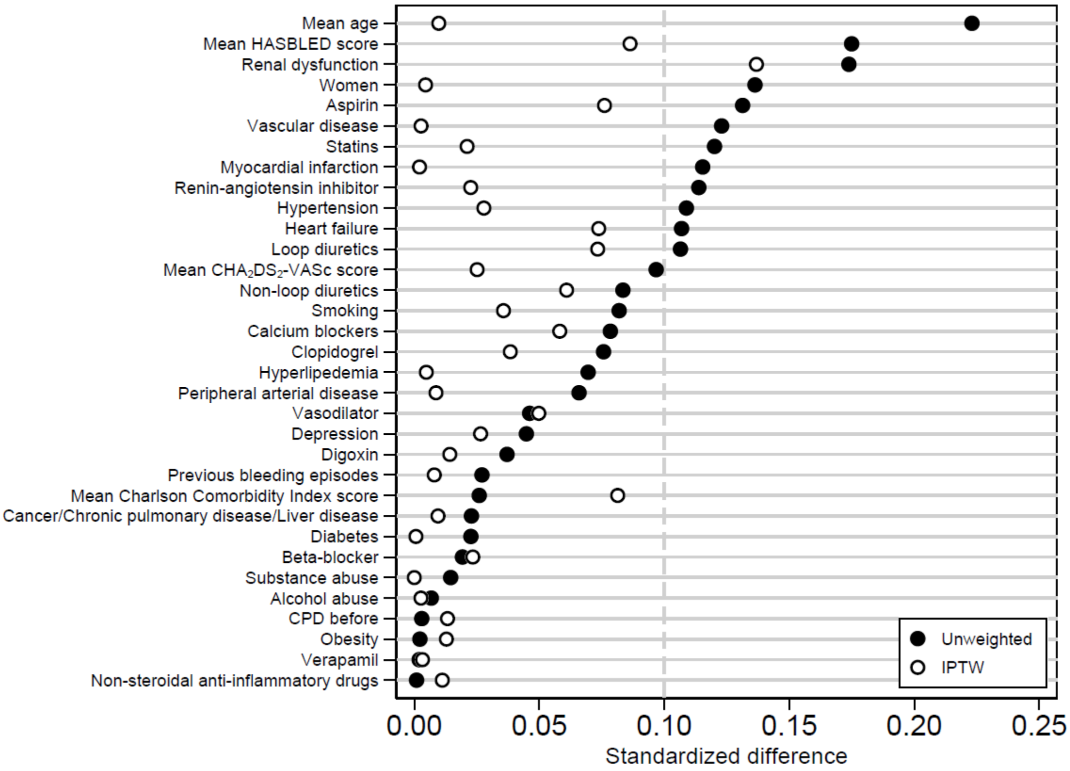

Figure S3. Propensity score distributions for warfarin and NOAC by age group. Overall

60-69 years **70-79 years** **80 years and older**

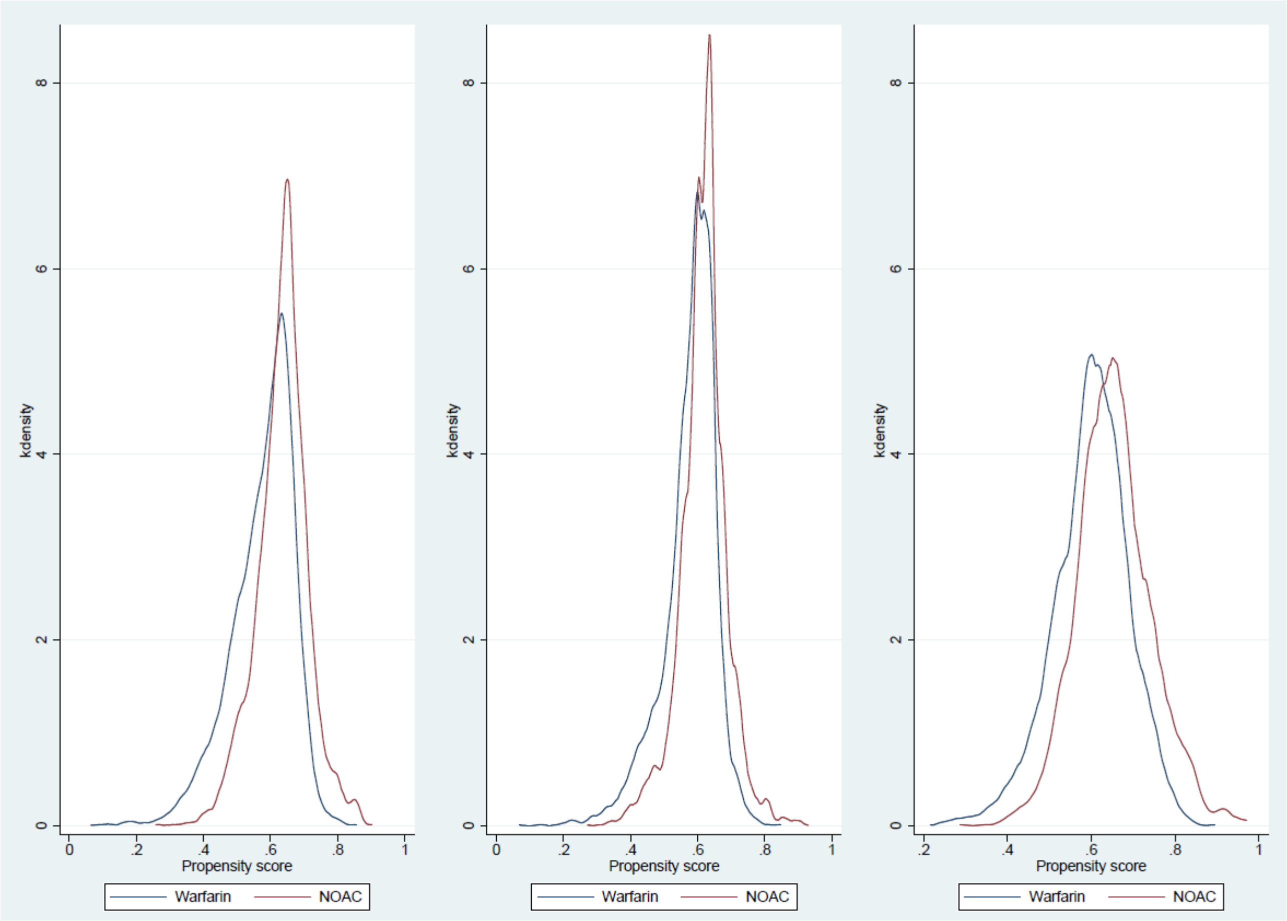

Standard dose NOAC

60-69 years **70-79 years** **80 years and older**

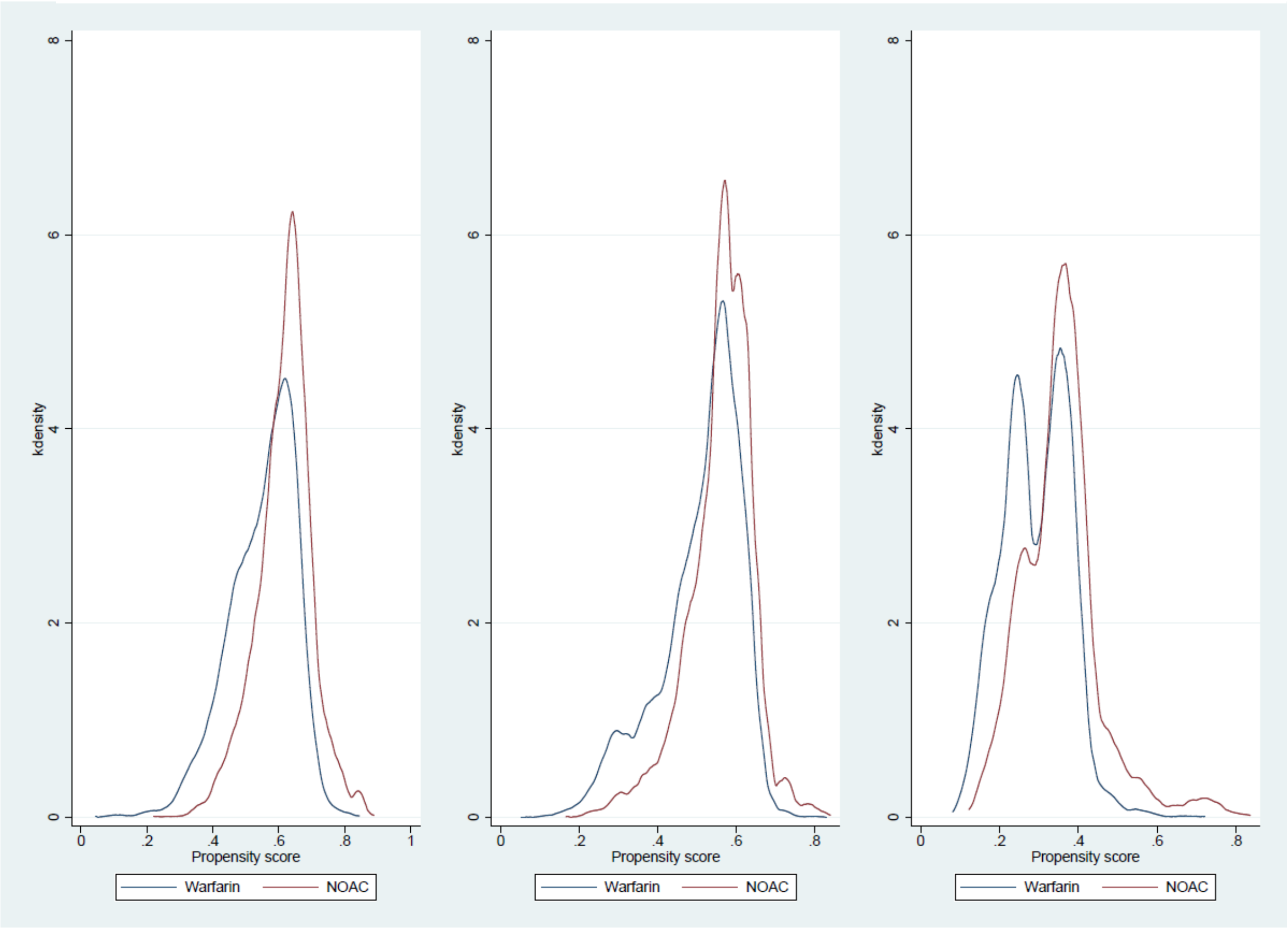

Reduced dose NOAC

60-69 years **70-79 years** **80 years and older**

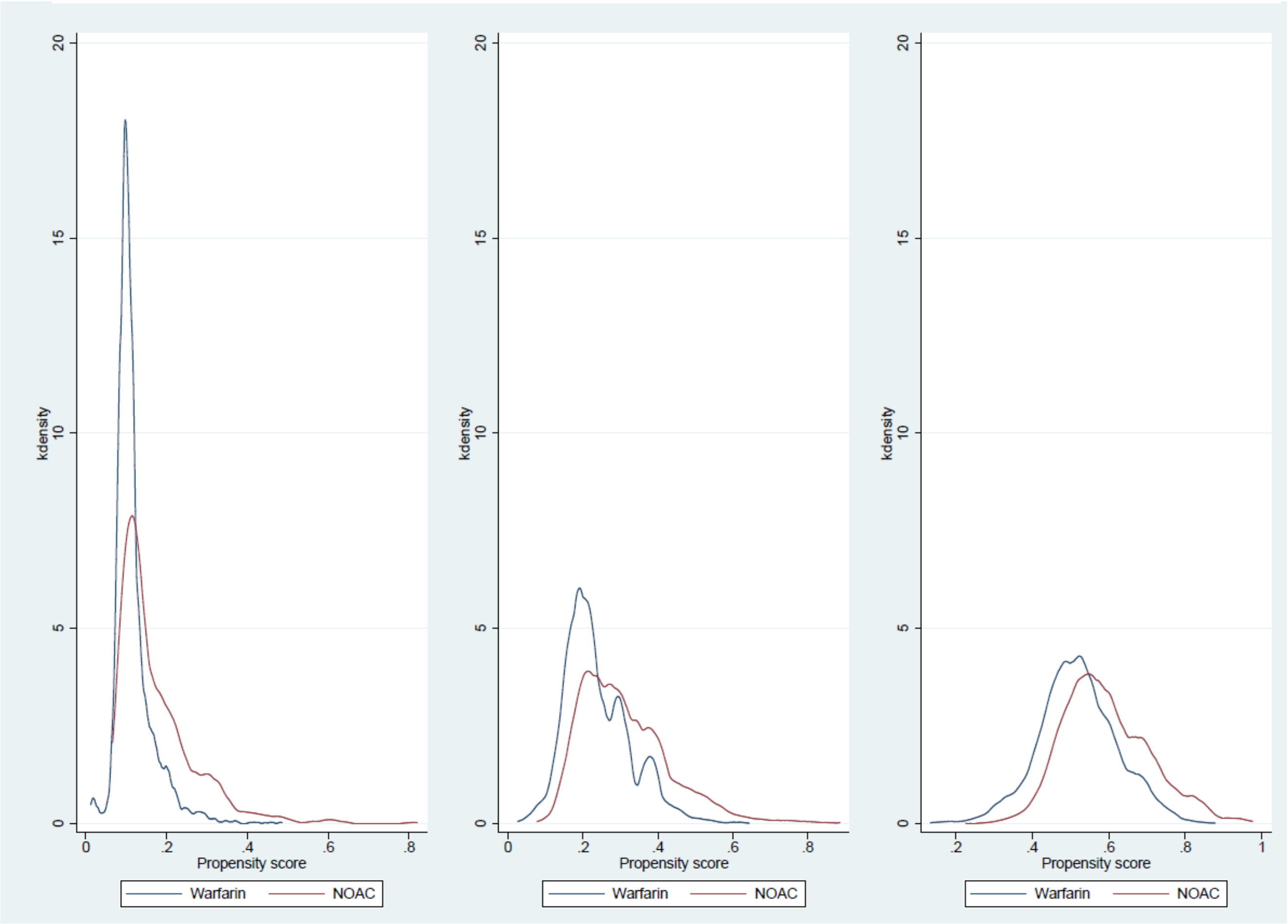

Supplement: Supplementary file 1 — Table S1. Definitions on Comorbidity and Concomitant Medication According to ICD‐10 Codes and ATC Codes Table S2. Risk Score Definitions Table S3. Definition of Charlson Comorbidity Index Table S4. Patient Characteristics by Age at Time of Index Oral Anticoagulant Prescription Claim and at the 180‐Day Landmark Before Propensity Weighting Table S5. Crude and Weighed HRs for the Association Between Exposure and Falsification End Points Figure S1. Change in patient characteristics by age from the time of index oral anticoagulant prescription claim (day 0) to the 180‐day landmark before propensity weighting visualized by signed standardized differences Figure S2. Plot of maximum of pairwise standardized differences for patient characteristics of nonvitamin K antagonist oral anticoagulant (NOAC) users and warfarin users at the 180‐day landmark stratified by age before and after applying propensity (inverse probability of treatment) weighting Figure S3. Propensity score distributions for warfarin and nonvitamin K antagonist oral anticoagulants (NOACs) by age group. [file JAH3-8-e011358-s001.pdf]
